# Gene expression profiling reveals U1 snRNA regulates cancer gene expression

**DOI:** 10.18632/oncotarget.22842

**Published:** 2017-12-01

**Authors:** Zhi Cheng, Yu Sun, Xiaoran Niu, Yingchun Shang, Jishou Ruan, Ze Chen, Shan Gao, Tao Zhang

**Affiliations:** ^1^ College of Life Sciences and Key Laboratory of Bioactive Materials Ministry of Education, Nankai University, Tianjin 300071, P.R. China; ^2^ Institute of Statistics, Nankai University, Tianjin 300071, P.R. China; ^3^ School of Mathematical Sciences, Nankai University, Tianjin 300071, P.R. China; ^4^ State Key Laboratory of Veterinary Etiological Biology and Key Laboratory of Veterinary Parasitology of Gansu Province, Lanzhou Veterinary Research Institute, Chinese Academy of Agricultural Science, Lanzhou, Gansu 730046, P.R. China

**Keywords:** U1 snRNA, over-expression, cancer, gene expression profiling, microarray

## Abstract

U1 small nuclear RNA (U1 snRNA), as one of the most abundant ncRNAs in human cells, plays an important role in splicing of pre-mRNAs. Compared to previous studies which have focused on the primary function of U1 snRNA and the neurodegenerative diseases caused by abnormalities of U1 snRNA, this study is to investigate how U1 snRNA over-expression affects the expression of mammal genes on a genome-wide scale. By comparing the gene expression profiles of U1 snRNA over-expressed cells with those of their controls using microarray experiments, 916 genes or loci were identified significantly Differentially Expressed (DE). These 595 up-regulated DE genes and 321 down-regulated DE genes were analyzed using annotations from GO categories and pathways from the KEGG database. As a result, three of 12 enriched pathways were well-known cancer pathways, while the other nine pathways were associated to cancers in previous studies. The further analysis of 73 genes involved in 12 pathways suggested that U1 snRNA could regulate cancer gene expression. The microarray data under the GEO Series accession number GSE84304 is available in the NCBI GEO database.

## INTRODUCTION

U1 small nuclear RNA (U1 snRNA) is one of the most abundant ncRNAs [1]. U1 snRNA has a length of 164 nt in human and its protein binding sites are highly conserved in insects and mammals [2]. The primary function of U1 snRNA is its involvement in the splicing of pre-mRNAs in nuclei. It has been known that snRNAs are synthesized in nuclei and transported to cytoplasm to retrieve other core spliceosome components, then return to nuclei for their functions [3]. U1 snRNA also functions in the regulation of transcription factors or RNA polymerase II, and maintaining telomeres.

Abnormalities of U1 snRNA can cause defects in pre-mRNA splicing, which are considered as a primary cause of human diseases [4]. In the year of 2013, Bai *et al.* discovered cytoplasmic aggregation of U1 snRNA with several U1 small nuclear Ribonucleoproteins (U1 snRNPs) in Alzheimer's Disease (AD), but not in other examined neurodegenerative diseases including Parkinson's Disease (PD) and Frontotemporal Lobar Degeneration (FTLD) [5]. Bai *et al.* also demonstrated that cytoplasmic aggregation of U1 snRNA and U1 snRNPs resulted in a loss of nuclear spliceosome activity, which altered the expression of Amyloid Precursor Protein (APP) and Amyloid Beta (Aβ) protein. APP contains a region that generates the Aβ protein, the aggregation of which has been considered as a main factor to cause AD. In the past 20 years, research of U1 snRNA has focused on its primary function, particularly related to neurodegenerative diseases caused by abnormalities of U1 snRNA. Besides the primary function, a non-canonical role for U1 snRNPs has been reported to protect pre-mRNAs from drastic premature termination by Premature Cleavage and Polyadenylation (PCPA) from cryptic Polyadenylation Signals (PASs) in introns [6]. PCPA yields shorter mRNAs using more proximal Alternative Polyadenylation (APA) sites, which has already been proved in activated immune, neuronal, and cancer cells [7]. Although previous studies concluded that Alternative Splicing (AS) and APA were deregulated and exploited by cancer cells to promote their growth and survival [8], the relationship between expression levels of U1 snRNA and cancers or cancer genes has not been investigated to the best of our knowledge.

In this study, we built a model of U1 snRNA over-expression in a rat cell line. Based on this model, we compared the gene expression profiles of U1 snRNA over-expressed cells with those of their controls to reach two research goals: 1) to study how U1 snRNA over-expression affects the expression of mammal genes on a genome-wide scale and identify the significantly Differentially Expressed (DE) genes; 2) to investigate the cellular effects caused by these DE genes. Mapping DE genes to Gene Ontology (GO) categories and Kyoto Encyclopedia of Genes and Genomes (KEGG) pathways, we found that two enriched GO terms and all the enriched KEGG pathways were associated to cancers. These results suggest that U1 snRNA regulates cancer gene expression, which has also been validated by quantitative PCR (qPCR) assays using human cell line, rat cell line and mouse tissues from animal experiments. Here, we report these results for the first time to help future studies of U1 snRNA in their functions in cancers or the development of new therapeutic targets for cancers.

## RESULTS

### U1 snRNA over-expression by transfection

**Figure 1:**
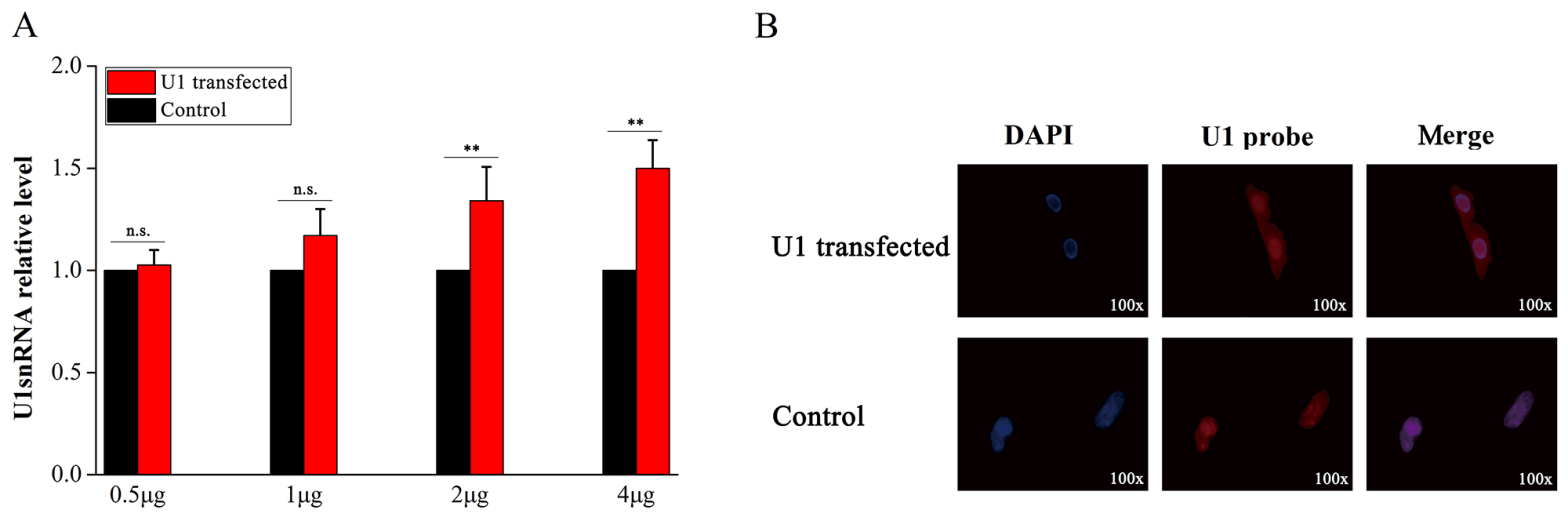
Validation of U1 snRNA over-expression in PC-12 cells Three samples in the treatment group were transfected with pSIREN-RetroQ plasmids containing the U1 snRNA sequences separately. The other three samples in the control group were transfected with pSIREN-RetroQ plasmids containing 5-bp polyA sequences. After the exogenous U1 snRNA genes expressed for 8 hours in the cells, qPCR and *in situ* hybridization were performed for model validation. **(A)** The expression levels of U1 snRNA in six samples were measured by qPCR and the expression levels in three control samples were normalized into 1. **(B)** 4-μg plasmids were used for transfection of each sample. Cell nuclei (in blue color) were stained with DAPI (in the left column). U1 snRNA were *in situ* hybridized with biotin-labeled LNA probes stained in red color (in the middle column).

Three U1-transfected samples in the treatment group and three samples in the control group were used to validate the model of U1 snRNA over-expression in PC-12 cells (Materials and methods). After the exogenous U1 snRNA genes expressed for 8 hours in the cells, qPCR and *in situ* hybridization were performed for model validation. The data obtained from qPCR assays showed a dose-dependent increase of U1 snRNA in U1-transfected samples, indicating that U1 snRNA had successfully been over-expressed in PC-12 cells (Figure 1A). U1 snRNA over-expression in all the samples was confirmed using immunofluorescent labeling with the 2,2,7-trimethylguanosine antibody following the procedure described in [3]. Since transfection with 5 μg plasmids or more resulted in cell death, *in situ* hybridization was performed to determine the locations of the U1 snRNA transcripts in cells using 4 μg plasmids for each sample. Fluorescence images indicated that U1 snRNA transcripts were located in nuclei of the control samples under normal conditions, while U1 snRNA transcripts had cytoplasmic accumulation in U1-transfected samples (Figure 1B).

### Differentially expressed genes and their GO analysis

Three U1-transfected samples (PC-12 cells) with 4 μg plasmids and three control samples were used for microarray experiments (Materials and methods). In total, 916 (about 4.42 % of 20,715) DNA probe sets were identified significantly Differentially Expressed (DE) between the treatment (U1-transfected) group and the control group. These probe sets represented 595 up-regulated and 321 down-regulated known genes or loci (Supplementary Table 1). These 916 significantly DE genes were annotated by Gene Ontology (GO) categories or terms in three GO domains (Supplementary Table 2), which were Molecular Function (MF), Cellular Component (CC) and Biological Process (BP). In the MF domain, highly represented categories were cytokine activity (GO:0005125), chemokine activity (GO:0008009), chemokine receptor binding (GO:0042379) and so on. The top three enriched categories in the CC domain were extracellular space (GO:0005615), extracellular region part (GO:0044421) and extracellular region (GO:0005576). In the BP domain, the top three categories were response to wounding (GO:0009611), regulation of phosphorylation (GO:0042325) and regulation of transferase activity (GO:0051338). Among these categories, tumor necrosis factor receptor superfamily binding (GO:0032813) and tumor necrosis factor receptor binding (GO:0005164) in the MF domain were associated to cancers.

### Pathway analysis of DE genes

The 916 DE genes were mapped to pathways from the Kyoto Encyclopedia of Genes and Genomes (KEGG) database and the enrichment analysis resulted in 12 enriched pathways in total (Supplementary Table 3). Among these 12 pathways (Table 1), three well-known cancer pathways were p53 signaling pathway (KEGG: rno04115), bladder cancer (KEGG: rno05219) and pathways in cancer (KEGG: rno05200). As for the other nine pathways, MAPK signaling pathway (KEGG: rno04010) [9], cell cycle pathway (KEGG: rno04110) [10], cytokine-cytokine receptor interaction pathway (KEGG: rno04060) [11], NOD-like receptor signaling pathway (KEGG: rno04621) [12], cytosolic DNA-sensing pathway (KEGG: rno04623) [13], RIG-I-like receptor signaling pathway (KEGG: rno04622) [14], Toll-like receptor signaling pathway (KEGG: rno04620) [15], chronic myeloid leukemia pathway (KEGG: rno05220) [16] and Hematopoietic cell lineage pathway (KEGG: rno04640) [17] had also been associated to cancers in previous studies. Among the total 73 genes involved in 12 enriched pathways, 56 and 17 were up- and down-regulated, respectively (Supplementary Table 3). These preliminary results suggest U1 snRNA regulates cancer gene expression.

**Table 1:** Enriched pathways induced by U1 snRNA over-expression

Pathway	Up-regulated	Down-regulated
rno04010:MAPK signaling pathway	Pak1, Nf1, Il1r2, Rras2, Dusp8, Nfkb1, Rapgef2, Fos, Traf2, Dusp5, Myc, Ddit3, Tgfb2, Tnf, Gadd45a, Gadd45g, Dusp1, Gadd45b	Pla2g2a, Tgfb1, Cd14, Map4k2, Cdc25b, Ntf4, Pla2g6, Fgf2, Ntrk2
rno04110:Cell cycle	Wee2, Cdkn1a, Smc1b, Mdm2, Cdk6, Cdkn2a, Myc, Tgfb2, Gadd45a, Gadd45g, Gadd45b	Tgfb1, Rbl2, Cdc6, Cdc25b, Ccne1
rno04060:Cytokine-cytokine receptor interaction	Il1r2, Tnfsf15, Il9r, Ccl5, Ifnb1, Cxcl2, Tnf, Il22ra2, Csf1, Cx3cl1, Cxcl10, Ltb, Tnfsf18, Il23r, Il6, Ccl4, Cxcl11	Tnfsf9, Il23a, Csf3
rno04621:NOD-like receptor signaling pathway	Ripk2, Cxcl1, Nfkb1, Birc3, Ccl5, Tnfaip3, Cxcl2, Tnf, Il6	Pycard
rno04115:p53 signaling pathway	Bid, Cdkn1a, Mdm2, Cdk6, Cdkn2a, Gadd45a, Gadd45g, Bbc3, Gadd45b	Ccne1
rno04623:Cytosolic DNA-sensing pathway	Nfkb1, Ccl5, Ifnb1, Cxcl10, Il6, Ccl4	Pycard
rno04622:RIG-I-like receptor signaling pathway	Ifih1, Nfkb1, Traf2, Ifnb1, Tnf, Dhx58, Isg15, Cxcl10	
rno04620:Toll-like receptor signaling pathway	Nfkb1, Fos, Tlr5, Ccl5, Ifnb1, Tnf, Cxcl10, Il6, Cxcl11	Cd14
rno05220:Chronic myeloid leukemia	Cdkn1a, Nfkb1, Mdm2, Runx1, Cdk6, Cdkn2a, Myc, Tgfb2	Tgfb1
rno04640:Hematopoietic cell lineage	Il1r2, Cd22, Il9r, Tnf, Csf1, Il6	Cd14, Csf3, Cd37
rno05219:Bladder cancer	Cdkn1a, Cdh1, Mdm2, Rassf1, Cdkn2a, Myc	
rno05200:Pathways in cancer	Bid, Cdkn1a, Cdh1, Nfkb1, Fos, Mdm2, Runx1, Hhip, Traf2, Rassf1, Cdk6, Birc3, Cdkn2a, Myc, Traf1, Tgfb2, Lamc2, Il6	Tgfb1, Ccne1, Fgf2

Pathways use data from the KEGG database. Gene names use HUGO gene symbols.

### Experimental validation and further analysis

**Figure 2:**
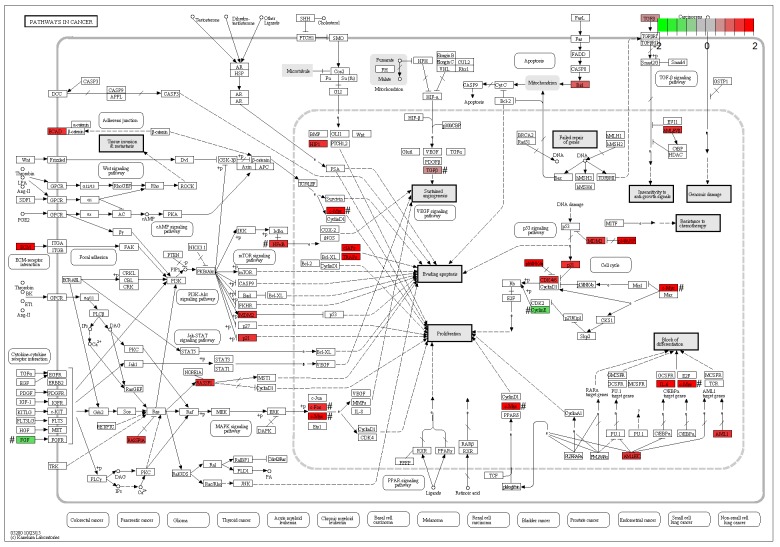
Pathways in cancer The figure of Pathways in cancer (KEGG: rno05200) was downloaded from the KEGG website. In this map, 21 DE genes included 18 up-regulated genes (Cdk6, Cdkn1a, Lamc2, Il6, Myc, Cdkn2a, Hhip, Traf2, Fos, Mdm2, Rassf1, Runx1, Bid, Traf1, Birc3, Nfkb1, Tgfb2 and Cdh1) in red color and three down-regulated genes (Ccne1, Fgf2 and Tgfb1) in green color. ^#^ The expression levels of six genes were confirmed by qPCR.

**Figure 3:**
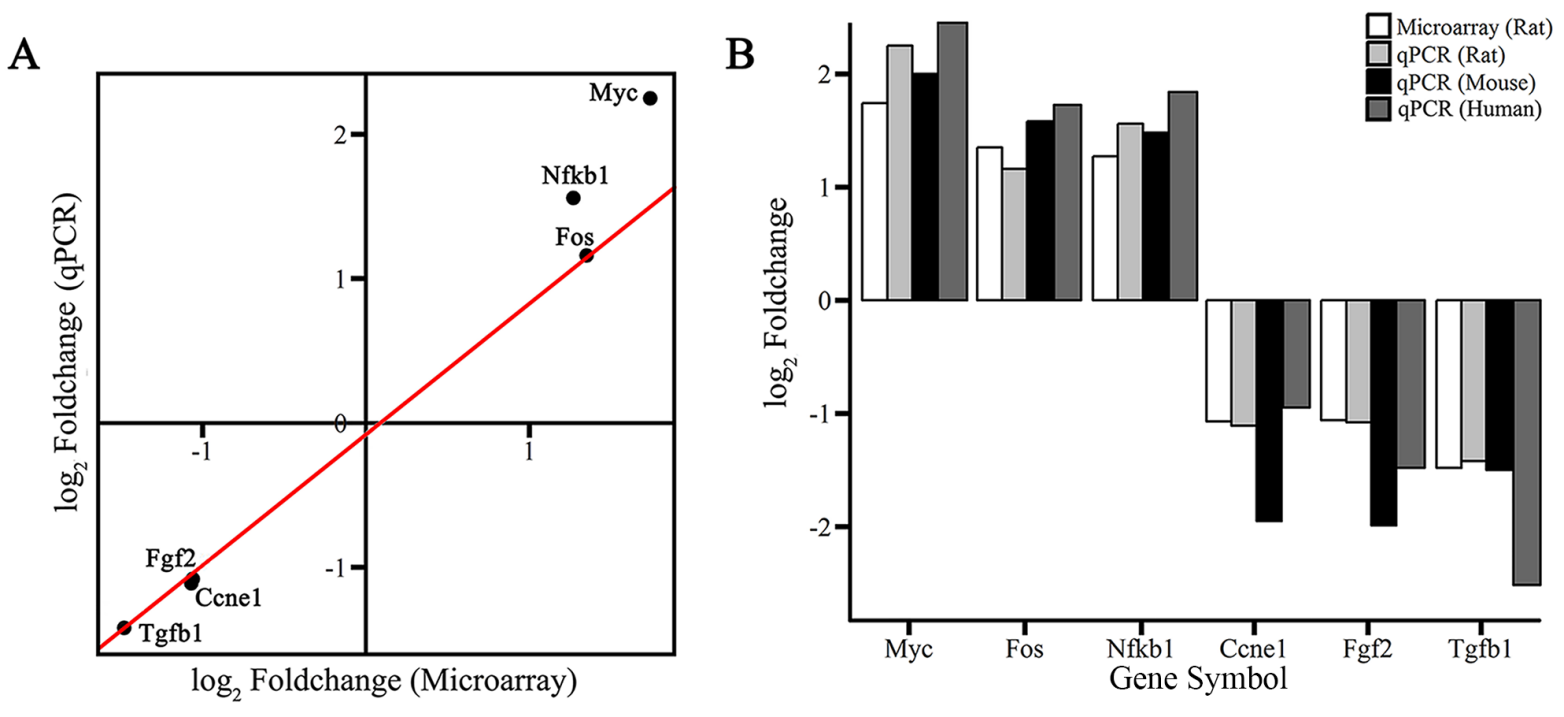
Confirmation of microarray results by qPCR The expression levels of three up-regulated DE genes (Myc, Fos and Nfkb1) and three down-regulated DE genes (Ccne1, Fgf2 and Tgfb1) were measured using qPCR. **(A)** Fold changes represent the average expression level of each gene in the treatment group (PC-12 cells) divided by that in the control group. A linear model was fit using the data of six genes. **(B)** Expression levels of three cell-apoptosis marker genes (Bax, Bcl2 and Casp3) and three cell-proliferation marker genes (Pcna, Mcm2 and Ki-67) were measured by qPCR assays using human cell line (Hela), rat cell line (PC-12) and mouse tissues (the hippocampus) from animal experiments.

To validate results from the microarray data analysis, the expression levels of six out of the total 21 DE genes (Figure 2) in pathways in cancer (KEGG: rno05200) were measured by qPCR assays using human cell line (Hela), rat cell line (PC-12) and mouse tissues (the hippocampus) from animal experiments (Materials and methods). The fold changes of six DE genes between the treatment and the control samples showed that Myc, Fos and Nfkb1 had been up-regulated and Ccne1, Fgf2 and Tgfb1 had been down-regulated, which were consistent with results from the microarray data analysis (Figure 3).

To investigate the cellular effects caused by U1 snRNA over-expression, we measured expression levels of three cell-apoptosis marker genes (Bax, Bcl2 and Casp3) and three cell-proliferation marker genes (Pcna, Mcm2 and Ki-67) by qPCR assays using human cell line (Hela), rat cell line (PC-12) and mouse tissues (the hippocampus) from animal experiments (Supplementary Table 4). Both three apoptosis markers and three proliferation markers were not significantly changed by U1 snRNA over-expression in PC-12 cells. Then, we performed U1 snRNA knockdown experiments using RNAi following the procedure described in [6] and the results showed a low Bax/Bcl2 ratio (~ 0.3) in PC-12 cells caused by U1 snRNA knockdown. It is well known that a low Bax/Bcl2 ratio (< 1) is against cell apoptosis, while a high Bax/Bcl2 ratio (>1) is in favor of cell apoptosis. U1 snRNA knockdown also caused significantly increased expression of Pcna, Mcm2 and Ki-67 by 2.95, 4.11 and 1.65 fold changes in PC-12 cells. The animal experiments showed a high Bax/Bcl2 ratio (~ 1.35) and significantly increased expression of Casp3 by two-fold change in mouse hippocampus tissues caused by U1 snRNA over-expression. These results suggested that U1 snRNA over-expression induced apoptosis in neural cells, which was consistent with the results from our previous study [18]. When it comes to the experimental results in Hela cells, the Bax/Bcl2 ratio reached 1.003, which suggested that U1 snRNA over-expression had not induced apoptosis in cancer cells. In addition, U1 snRNA over-expression caused significantly increased expression of Pcna, Mcm2 and Ki-67 by 10.44, 12.8 and 2.13 fold changes in Hela cells, which suggested that U1 snRNA over-expression promoted proliferation in cancer cells.

## MATERIALS AND METHODS

### Transfection of U1 snRNA into cell nuclei

PC-12 cells (rat) were cultured in RPMI-1640 medium containing 10% fetal bovine serum. The medium was changed every two days and the cells were passaged every four days. To perform the transfection of U1 snRNA, PC-12 cells were sampled for six times (about 2×10^6^ cells per sample), then trypsinized, washed once with PBS and resuspended in 100 μL Nucleofector Solution (Lonza-Amaxa, German). Three samples (the treatment group) were transfected with 0.5, 1, 2, 4 and 5 μg pSIREN-RetroQ plasmids [7] (Clontech, USA) containing the 168-bp U1 snRNA sequences (Genbank: V01266.1) separately. The other three samples (the control group) were transfected with empty pSIREN-RetroQ plasmids containing 5-bp polyA sequences. Nucleus transfection into six samples was performed by electroporation using a Lonza-Amaxa Nucleofector Pulser with the program setting U-029 and then cultured in 2.5 mL RPMI-1640 medium for 8 h. Hela cells (human) were cultured and transfected following the same procedure described in [6], but using the 164-bp human U1 snRNA sequences (RefSeq: NR_004430.2).

U1 snRNA transcripts in PC-12 cells were *in situ* hybridized with 25-bp biotin-labeled LNA probes 5′-CATGGTATCTCCCTGCCAGGTAAGT-3′ (Exiqon, Denmark) for model validation following the procedure described below. For each sample, the hybridization buffer (50% v/v formamide, 2X SSC, 50 mM sodium phosphate with pH 7 and 10% dextran sulfate) was mixed with 10 nM probes and then added into the cells at 55°C for 12 h in a humidified chamber. After hybridization, cells were washed in 2X SSC, then incubated in 0.1% Triton at 4°C for half an hour and washed in 2X SSC. Alexa Fluor 594 streptavidin conjugate (Yeasen, China) was added into the cells to stain biotin-labeled LNA probes into red color. Cells were washed in 4X SSC at 4°C and then washed in 2X SSC, 1X SSC and PBS subsequently at room temperature. Cell nuclei were stained by DAPI into blue color and examined using a fluorescence microscope (Olympus, Japan).

### Microarray experiments

The second generation of PC-12 cells were passaged and also sampled for six times (about 2×10^6^ cells per sample) for microarray experiments. Following the same procedure described above, three samples in the treatment group and the other three samples in the control group were transfected by pSIREN-RetroQ plasmids containing the 168-bp U1 snRNA sequences (Genbank: V01266.1) and 5-bp polyA sequences, respectively. Total RNA was isolated from six samples using RNAiso Plus Reagent (TaKaRa, Japan) and then processed separately. The cDNA synthesis and antisense RNA (aRNA) amplification were performed using Amino Allyl MessageAmp II aRNA Amplification Kit (Ambion, USA). The modified nucleotide, 5-(3-aminoallyl)-UTP (aaUTP), was incorporated into the amplified aRNA during the *in vitro* transcription with T7 RNA Polymerase to form amino allyl-aRNA (aa-aRNA). The aa-aRNA was readily labeled with amine-reactive N-hydroxysuccinimide (NHS) ester of fluorescent dyes (Cy5-NHS). Finally, six samples were hybridized to six chips of Rat OneArray Plus (Phalanx Biotech, China) separately. The Rat OneArray Plus chip contains 20,715 DNA probe sets with the length of 60 bp.

### Animal experiments

To construct U1 snRNA over-expression lentiviruses, DNA regions of U1 snRNA (Genbank: J00645.1) were amplified using total DNA from mouse hippocampus tissues. The amplified DNA fragments were subjected to electrophoresis in 1% agarose gel, recovered using MiniBEST Agarose Gel DNA Extraction Kit v4.0 (TaKaRa, Japan), ligated into the pLVX-shRNA1 plasmids using In-fusion HD Cloning Kit (Clontech, USA) and transfected into *E. coli* JM109 competent cells (TaKaRa, Japan). After transfection, *E. coli* cells were being shaken at 150 rpm for 16 h, followed by extraction of plasmids and preparation of lentiviruses using Lenti-X HTX Packaging System (Clontech, USA), following the protocol described by the manufacturer.

After their habituation to the environment for one week, six 9-week-old C57BL/6J male mice were randomly divided into the treatment group (n=3) and the control group (n=3). These mice were weighed and then anesthetized (0.6 g/kg bodyweight) by intraperitoneal injection of chloral hydrate (Sigma-Aldrich, USA). Afterwards, they were placed in a SN-3 stereotaxic frame (Narishige, Japan). For each mouse in the treatment group, 1 μL U1-packged lentiviruses were injected into lateral ventricles (0.1 mm posterior to the bregma, 0.9 mm lateral to midline, 2.0 mm ventral below the dura) of their brains at a flow rate of 0.5 μL/min using a 10-μL syringe (Hamilton, USA). Empty lentiviruses were injected into the mice in the control group. After injection, the needle was kept in place for 5 min before it was slowly withdrawn. Two month later, six mice were decapitated to obtain their hippocampus tissues for qPCR assays.

### Quantitative PCR assays

The third generation of PC-12 cells were passaged for qPCR assays of 12 genes (Myc, Fos, Nfkb1, Ccne1, Fgf2, Tgfb1, Bax, Bcl2, Casp3, Pcna, Mcm2 and Ki-67). Hela cells and mouse tissues (the hippocampus) from animal experiments described above were also used for qPCR assays. For each gene, three U1-transfected and three control samples (about 2×10^6^ cells per sample) were tested for relative quantification. U1 snRNA transfection, RNA extraction, cDNA synthesis and cDNA amplification followed the same procedure described below. Total RNA was isolated from samples using RNAiso Plus Reagent (TaKaRa, Japan) and then processed separately. The cDNA of U1 snRNA was synthesized by Mir-X miRNA First-Strand Synthesis Kit (Clontech, USA). The cDNA product was amplified by qPCR (Eppendorf, German) using U6 snRNA as internal control under specific reaction conditions (Supplementary Table 4).

### Data analysis

Six chips were scanned into images in TIF format using the Agilent G2505C Microarray Scanner System (Agilent Technology, USA). Images were read into gene expression data (numerical values) in GPR format using the software GenePix Pro v4.1.1.44 (Axon Instruments, CA, USA). The gene expression data were normalized and further analyzed to determine the significantly Differentially Expressed (DE) genes between the treatment group and the control group using the software Rosetta Resolver System v7.2 (Rosetta Biosoftware, USA). All the DE genes satisfied both the P-value < 0.05 and the absolute log2 fold-change >= 1. DNA probes were annotated by the software Annotation ROA2 r1.0 (Phalanx Biotech, China) with two databases (NCBI RefSeq r65 and Ensembl r76 cDNA sequences Rnor_5.0 annotations). Statistical computation and plotting were performed using the software R v2.15.3 with the Bioconductor packages [19]. The annotations and enrichment analyses by GO terms and KEGG pathways were performed using Gene Set Enrichment Analysis (GSEA) (http://software.broadinstitute.org/gsea/index.jsp).

## CONCLUSIONS

In this study, we investigated how U1 snRNA over-expression affected the expression of mammal genes on a genome-wide scale using PC-12 cells. Based on the results from differential expression analysis, we reported for the first time that U1 snRNA could regulate cancer gene expression. Further experiments and analysis suggested that U1 snRNA over-expression could induce apoptosis in neural cells and proliferation in cancer cells, while U1 snRNA knockdown could induce proliferation in neural cells and apoptosis in cancer cells.

## SUPPLEMENTARY MATERIALS AND TABLES










