# Supplementary material for: Gene expression profiling reveals U1 snRNA regulates cancer gene expression
Source: Oncotarget. 2017 Dec 1;8(68):112867–74. doi: 10.18632/oncotarget.22842 (PMC5762557; doi:10.18632/oncotarget.22842)
Supplement: Supplementary file 1 [file oncotarget-08-112867-s001.pdf]

Gene expression profiling reveals u1 snrna regulates cancer gene expression

SUPPLEMENTARY MATERIALS

Supplementary Table 1: Differentially Expressed (DE) genes between U1 over-expression samples and their controls.

See Supplementary File 1

Supplementary Table 2: Gene Ontology (GO) annotation and enrichment.

See Supplementary File 2

Supplementary Table 3: KEGG pathway annotation and enrichment.

See Supplementary File 3

Supplementary Table 4: All qPCR results with the primer information.

Supplementary File 4
